# Bilateral Ganglion Cysts of the Ligamentum Flavum in the Cervical Spine Causing a Progressive Cervical Radiculomyelopathy and Literature Review

**DOI:** 10.1155/2017/3953641

**Published:** 2017-07-31

**Authors:** Juneki Kim, Jin-gyu Choi, Byung-chul Son

**Affiliations:** ^1^Department of Neurosurgery, Seoul St. Mary's Hospital, College of Medicine, The Catholic University of Korea, Seoul, Republic of Korea; ^2^Catholic Neuroscience Institute, College of Medicine, The Catholic University of Korea, Seoul, Republic of Korea

## Abstract

Here we report a unique case of bilateral ganglion cysts originating from the ligamentum flavum in the cervical spine. Degenerative cysts of the ligamentum flavum are rare lesions, and most had been reported in the lumbar spine. Its occurrence in the cervical spine is extremely rare: only eight have been reported. A 66-year-old male patient presented with progressive paraparesis, pain, and paresthesia in his bilateral T1 dermatomes that had lasted for three weeks. Magnetic resonance imaging of the cervical spine demonstrated a well-demarcated cystic lesion in the bilateral dorsolateral aspects of the C7/T1 segment and significant compression of the cervical cord. All case reports of ganglion cysts of the cervical ligamentum flavum including the present one showed characteristic symptoms and signs of myelopathy such as paraparesis or quadriparesis associated with varying degrees of paresthesia or pain in the upper extremities. Ganglion cysts of the cervical ligamentum flavum are considered a cause of cervical radiculomyelopathy due to cervical intraspinal cystic lesions. Bilateral occurrence and associated subluxation of the involved cervical segments again support the degenerative pathogenesis of ganglion cysts of the ligamentum flavum in the cervical spine.

## 1. Introduction 

Ganglion cysts of the ligamentum flavum are uncommon degenerative spinal lesions that are mostly encountered in the lumbar spine [1–6]. Cervical localization is rare and may cause severe myelopathy [7]. These cysts accompany degenerative changes of the spine and can be differentiated from synovial and other degenerative spinal cysts based on location and histopathological features [7]. To our knowledge, only five cases have been reported, and all were associated with myelopathy [7–11]. Here, we report a case of bilateral ganglion cyst of the ligamentum flavum in the cervical spine that presented with gradual paraparesis along with bilateral cervical radicular pain.

## 2. Case Presentation

A 66-year-old male patient, usually in good health, presented progressive paraparesis of three weeks' duration. Three months prior to admission, he had worked hard for four to five hours a day for three months. He developed posterior neck pain and was treated several times with acupuncture. Three weeks earlier, the left leg had lost strength, and his right leg began to flex within a week. Eventually, he could not stand by himself due to the gradual progression of the paraparesis; he also felt paresthesia and pain on his bilateral medial upper arm. No urinary incontinence developed. He was admitted to a hospital and had magnetic resonance imaging (MRI) of the cervical spine and was referred for further evaluation.

**Figure 1:**
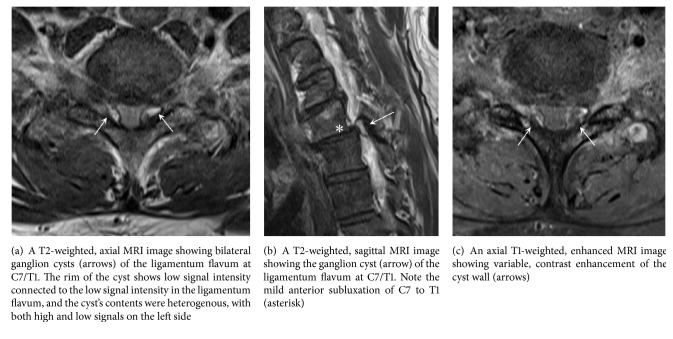
Magnetic resonance imaging (MRI) findings of bilateral ganglion cysts of the ligamentum flavum at C7/T1.

Neurologic examination showed paraparesis and ataxia. Decreased motor strength, mainly of the quadriceps and adductors, was noted in his lower extremities, 4/5 on his right side and 3/5 on the left. He had more pronounced deep tendon reflex in his left patellar than in his right one. His upper extremities showed no weakness, but he had pain associated with paresthesia and hypesthesia in bilateral C8 dermatomes. We observed no urinary difficulty. MRI of the cervical spine revealed bilateral extradural cystic masses that compromised the dural sac at the level of C7/T1 (Figure 1). The lesion was isointense in T1-weighted images and showed a hyperintense core with a peripheral hypodense ring in T2-weighted images. The wall of the cyst showed strong enhancement with gadolinium. We observed mild subluxation of C7 over T1 in the sagittal T2-weighted MRI and found prominent degenerative osteoarthritic changes in the bone scan. Considering the progressive neurologic deficits, we planned surgical treatment.

**Figure 2:**
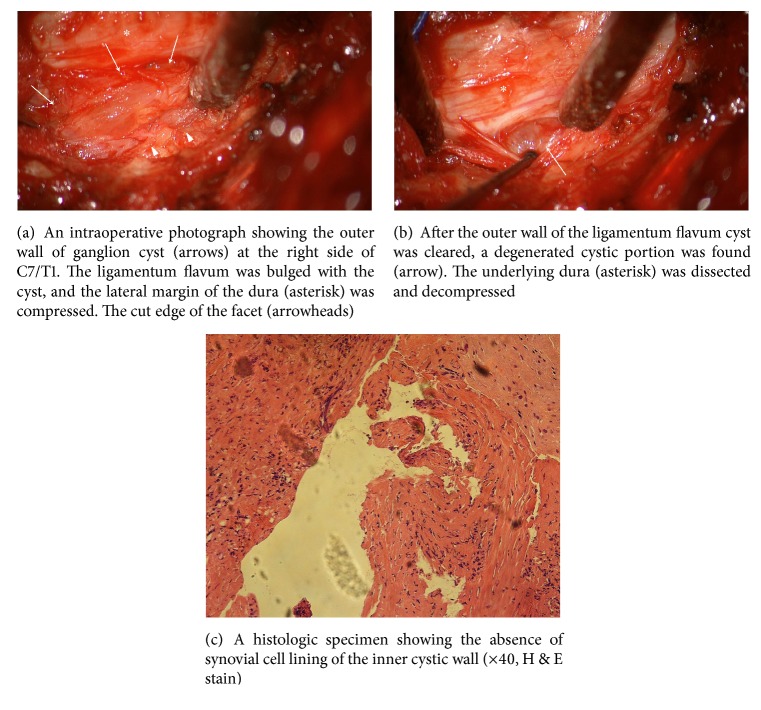
Intraoperative photograph showing the ganglion cyst of the ligamentum flavum.

After a laminectomy of C7 and small medial facetectomy of C7/T1, we removed a hypertrophied ligamentum flavum and exposed the underlying dura. We found a fibrotic, extradural cyst on the internal aspect of the ligamentum flavum (Figure 2) that was embedded within the inner aspect of the ligamentum flavum with no connection with the facet joint or dura mater. When we violated the cyst wall, we found thick, mucous fluid. The cyst wall adhered densely to the underlying, lateral margin of the dura, and we carefully dissected the adhesion under microscopy. After we completely removed the bilateral cysts of the ligamentum flavum and the adjacent hypertrophied ligamentum flavum, we found the dura and the bilateral C8 root to be decompressed.

The postoperative course was uneventful. Immediately after the operation, the patient's severe pain and paresthesia of the bilateral medial upper arm corresponding to the T1 dermatome were alleviated. Although he experienced immediate functional improvement in both legs, the weakness in both legs improved only slowly over the six months following the operation. There was no weakness or sensory deficit in his arms and legs at the one-year postoperative follow-up and no neck pain or radiological instability.

## 3. Discussion

### 3.1. Pathogenesis of Ganglion Cysts of the Ligamentum Flavum in the Cervical Spine

Intraspinal degenerative cysts are rare and usually located in the lumbar spine [1–6]. Because the joint capsule is often considered to be the origin of these lesions, they are called juxtafacet cysts to indicate both synovial and ganglion cysts [12, 13]. Owing to their similar locations and cystic contents, the terms “spinal synovial cyst” and “spinal ganglion cyst” have been used interchangeably [6]. Initially, the suggested difference between synovial and ganglion cysts was that the former often contain clear and serous fluid whereas the latter contain gelatinous, highly viscous fluid [12]. However, differentiation between them is only possible with pathological findings [6]. Synovial cysts are lined with pseudostratified columnar cells, whereas ganglion cysts have no synovial cell lining and no communication with the joint cavity [6, 9, 10, 13, 14]. In the present case, the cysts did not communicate with the facet joint but were instead imbedded in the ligamentum flavum intraoperatively, and we found no synovial lining on microscopic examination. Therefore, we made the diagnosis of ganglion cyst.

It has been suggested that ganglion cysts are caused by myxoid degeneration and cystic softening of the connective tissue of the joint capsule or tendon sheath as a result of degenerative process or trauma [7]. The pathogenesis of degeneration of the ligamentum flavum is still unclear, but it can be considered in the context of degenerative change [7]. Aging and repeated microtrauma due to spinal motion lead to degenerative changes including loss of elastic fibers, thickening with chondrocyte proliferation and calcifications, and formation of collagen fibers [6]. Loss of elasticity predisposes the ligamentum flavum to mechanical stress injury, resulting in scar remodeling and ganglion cyst formation [6]. The current case is unique because of the bilateral occurrence of the ligamentum flavum ganglion cyst and associated mild subluxation. We think that bilateral occurrence and associated subluxation further support the pathogenesis of degenerative changes involving the ligamentum flavum. Indeed, in the literature, all of the case reports involved people over age 60 (Table 1).

### 3.2. Symptoms, Diagnosis, and Treatment of Ganglion Cysts in the Cervical Ligamentum Flavum

Symptoms and signs of myelopathy with or without cervical radiculopathy association are the most common presenting symptoms in symptomatic ganglion cysts of the ligamentum flavum in the cervical spine. In our review of the literature regarding theses cysts, six of seven reported cases (86%), including the current case, showed paraparesis or quadriparesis owing to their location within the narrow cervical spinal canal. Most symptoms and signs of myelopathy are gradual and insidious (Table 1). However, an occurrence of sudden Brown-Sequard syndrome within three hours due to a ganglion cyst of the cervical ligamentum flavum has been reported [10]. Although the symptomatic cervical ligamentum flavum ganglion cysts in the literature have been small (8 to 15 mm in diameter), all reported cases showed characteristic symptoms and signs of myelopathy: gait disturbance and paraparesis [15–17].

MRI is the imaging study of choice in the diagnosis of ganglion cysts in the cervical spine, although histopathologic examination is needed for definitive diagnosis [7]. The MRI findings of the ganglion cysts are characteristic; the cyst contents are hypointense on T1-weighted images and hyperintense on T2-weighted images [6, 7, 9, 10, 14]. The peripheral rims of the cysts are hypointense on T2-weighted images with gadolinium enhancement. The treatment for symptomatic ganglion cysts of the ligamentum flavum in the cervical spine is surgery. All reported cases of ganglion cervical spine cysts caused serious neurologic deficits, and surgical treatment universally resulted in neurologic improvement.

Surgical excision of ganglion cysts of the cervical spine with posterior laminectomy appears to be a straightforward for decompressing the spinal cord, removing the ganglion cysts, addressing the connection to the facet joint, and taking histologic specimens for definitive diagnosis. All case reports regarding symptomatic ganglion cysts of the cervical ligamentum flavum adopted surgical excision via laminectomy, and the prognosis of surgical treatment is favorable. Although some degree of adhesion between the dura and the ganglion cyst was always mentioned, no surgical morbidity or neurologic compromise was reported. Gradual recovery of the symptoms and signs of radiculomyelopathy appear to occur within 6 to 12 months postoperatively.

## 4. Conclusions

We here report a very rare case of bilateral ganglion cysts of the cervical ligamentum flavum that presented with progressive myelopathy and radiculopathy. The characteristic bilateral occurrence and associated cervical subluxation at the involved segment supported the degenerative pathophysiology in the cyst.

## Figures and Tables

**Table 1:** Summary of the reported cases of ganglion cysts of the ligamentum flavum in the cervical spine.

Author/year	Number of cases	Age/sex	Presenting symptom/signs	Location	Diagnostic modality	Treatment	Prognosis follow-up period	Associated condition
Takano et al., 1992	1	72/m	Spastic paraparesis, 1 yr bilat. C6 paresthesia	C3/4, lt	MRI, OR, histology connection (−)	C3–6 laminectomy	Rapid recovery unknown F-U	
Yamamoto et al., 2001	2	81/m	Progr. paraparesis, 1 mo neck pain, bilat. arm leg paresthesia	C3/4, lt	MRI, OR, histology connection (−)	Laminectomy C3–7 laminoplasty	Improved, 2 mos	
		65/m	Spastic quadriparesis rt arm pain	C3/4, rt	MRI, M-CT connection (−)	Laminectomy C3–7 laminoplasty	Improved, 2 mos	
Shima et al., 2002	1	66/m	Paraparesis and numbness	C7/T1, rt	MRI, OR, histology connection (−)	Laminoplasty C3–6	Complete recovery, 9 mos	
Chenng et al., 2006	1	58/m	Sudden Brown-Sequard synd. lt-sided hemiparesis rt-sided hemianalgesia below T4	C6/7, lt	MRI, OR, histology connection (+)	Laminectomy C6-7	Complete recovery, 4 mos	CRF
Yahara et al., 2009	1	63/f	Myelopathy below C5, bilat. hand paresthesia	C4/5, lt	MRI, OR, histology connection (−)	Laminectomy C4/5 instrumentation, fusion	Complete recovery, 1 yr	RA 15 yrs C4/5 instability
Muzii et al., 2010	1	60/m	Progr. paraparesis, 1 yr ataxia, hyperreflexia	C4/5 midline	MRI, OR, histology connection (−)	C4/5 laminectomy	Complete recovery, 1 yr mild spastic gait	
Brotis et al., 2012	1	82/f	Progr. quadriparesis, 3 mos bilat. arm paresthesia, neck pain	C3/4, lt	MRI, OR, histology connection (−)	Laminectomy C3	Complete recovery, 6 mos	HBP, DM hypothyroidism
Current case, 2017	1	66/m	Paraparesis, 3-week bilat. T1 paresthesia, pain	C7/T1, bilat.	MRI, OR, histology connection (−)	Laminectomy C7	Complete recovery, 12 mos	C7/T1 subluxation

Bilat.: bilateral, CRF: chronic renal failure, DM: diabetes mellitus, HBP: hypertension, lt: left, mos: months, OR: operation, progr.: progressive, RA: rheumatoid arthritis, and rt: right. Connection (−)/(+); presence/absence of communication to the facet joint.
